# Major Postoperative Complications and Survival After Lung Cancer Resection in Patients Aged ≥80 Years: Risk Factor Analysis

**DOI:** 10.3390/curroncol33070405

**Published:** 2026-07-07

**Authors:** Fuad Damirov, Junli Ke, Javad Karimbayli, Mircea G. Stoleriu, Sascha Dreher, Enole Boedeker, Sibylle Gerz, Rudolf A. Hatz, Gerhard Preissler

**Affiliations:** 1 Department of Thoracic Surgery, Ludwig Maximilian University of Munich (LMU), 81377 Munich, Germany; 2 Department of Thoracic Surgery, Robert Bosch Pulmonary Center Stuttgart, Robert Bosch Hospital, Bosch Health Campus, 70376 Stuttgart, Germany; 3 Division of Molecular Oncology, Centro di Riferimento Oncologico (CRO) of Aviano, IRCCS, National Cancer Institute, 33081 Aviano, Italy; 4 Thumbay Research Institute for Precision Medicine, Gulf Medical University, Ajman 4184, United Arab Emirates; 5 Institute for Lung Health and Immunity and Comprehensive Pneumology Center, Helmholtz Center Munich, 81377 Munich, Germany

**Keywords:** lung cancer, thoracic surgery, postoperative complications, older patients, serum albumin, transfusion, survival

## Abstract

This study aimed to identify risk factors for major postoperative complications (TMM) and their impact on overall survival in patients aged ≥80 years undergoing lung cancer surgery, a group at increased risk of morbidity. A total of 88 patients aged ≥80 years who underwent anatomical lung resections were retrospectively analyzed. Postoperative complications were classified into minor or no complications (TMM 0–2) and major (TMM ≥ 3) complications. Logistic regression analysis was performed to identify independent predictors of major complications. Kaplan–Meier estimates were used to measure overall survival. Major complications occurred in 24 patients (27.3%). Multivariable regression analysis identified perioperative blood transfusion as an independent risk factor for major complications (*p* = 0.0009). Patients with major complications exhibited significantly reduced overall survival (*p* = 0.0009). Subgroup analysis revealed that patients who underwent minimally invasive surgery had significantly better survival (*p* = 0.01). Moreover, perioperative transfusion correlated with diminished overall survival (*p* = 0.0027). In patients aged ≥80 years undergoing lung resections, the occurrence of major postoperative complications is associated with significantly impaired survival. Minimally invasive surgical approaches and blood conservation strategies may mitigate complications and enhance long-term outcomes in this high-risk group.

## Simple Summary

Lung cancer surgery in patients aged 80 years and older is becoming more common, but it carries a higher risk of complications. This study examined which factors increase the likelihood of serious complications after surgery and how these complications affect long-term survival. We found that patients who developed major complications had significantly worse survival outcomes. Blood transfusions during or after surgery were strongly linked to these complications and poorer survival. In contrast, minimally invasive surgical techniques were associated with better outcomes. In addition, low blood protein levels before surgery were an important indicator of higher risk and reduced survival. These findings highlight the importance of careful patient selection, optimization of health before surgery, and the use of less invasive techniques. Improving these aspects of care may help reduce complications and improve long-term survival in older patients undergoing lung cancer surgery.

## 1. Introduction

Lung cancer remains the leading cause of cancer-related mortality, worldwide accounting for more deaths than prostate, breast and colorectal cancers combined [1,2]. Its incidence increases with age, with a marked rise observed in patients older than 80 years [3,4]. Surgical resection remains the only potentially true curative treatment for early-stage non-small cell lung cancer (NSCLC) [5]. Although anatomical lung resection remains the standard curative treatment for medically operable patients with early-stage NSCLC, alternative treatment strategies have emerged in recent years. Notably, Kleber et al. demonstrated comparable efficacy of stereotactic body radiotherapy (SBRT) in selected patients with early-stage lung cancer, highlighting the importance of individualized treatment decisions within a multidisciplinary setting [6]. However, decision-making in patients aged ≥80 years is particularly challenging due to reduced physiological reserve, multiple comorbidities, and an increased risk of postoperative complications and mortality [7,8]. At the same time, advances in surgical techniques and perioperative management have expanded the indications for lung cancer resection in this growing patient population [9,10,11,12].

Recent evidence suggests that, in carefully selected patients aged ≥80 years, surgical treatment can achieve favorable long-term survival and quality-of-life outcomes comparable to those of younger patients, despite higher rates of postoperative complications [5]. Nevertheless, elderly patients are often underrepresented in clinical trials, and data specifically addressing risk factors for major complications and their impact on long-term outcomes remain limited.

Postoperative complications play a crucial role in determining surgical outcomes, particularly in older patients. While several studies have identified preoperative functional parameters, such as pulmonary function tests and exercise capacity, as predictors of postoperative morbidity, these findings are largely derived from mixed-age populations and may not fully apply to patients aged ≥80 years [13,14,15,16,17]. Moreover, the severity of complications and their impact on long-term survival are not always adequately captured by conventional classification systems.

The Thoracic Morbidity and Mortality (TMM) classification provides a standardized and procedure-specific framework for grading postoperative complications in thoracic surgery, allowing for a more precise assessment of their clinical relevance [18]. However, its application in very old patients undergoing lung cancer surgery has not been sufficiently explored. Therefore, the aim of this study was to identify independent clinical and perioperative risk factors for major postoperative complications (TMM ≥ 3) following anatomical lung cancer resection in patients aged ≥80 years and to evaluate their impact on overall survival. In addition, perioperative outcomes, including morbidity, mortality, and length of hospital stay, were assessed to better define surgical risk in this high-risk population.

## 2. Materials and Methods

### 2.1. Study Population

This retrospective single-center cohort study was approved by the Ethics Committee of Ludwig Maximilian University of Munich (LMU), Germany (approval number 24-0114). The study was conducted in accordance with the Declaration of Helsinki and followed the recommendations of the STROBE statement for observational research. Patients were treated at the Department of Thoracic Surgery, Robert Bosch Hospital (Stuttgart, Germany), between 1 January 2014, and 31 December 2023. Eligible participants were aged 80 years or older and underwent anatomical pulmonary resection for a resectable primary lung malignancy. Surgical procedures included segmentectomy, lobectomy, bilobectomy, and pneumonectomy, with the diagnosis of lung cancer confirmed by intraoperative histopathological examination. Patients younger than 80 years, those undergoing non-anatomical resections, palliative procedures, or receiving neoadjuvant therapy were excluded from the analysis. Patients who received neoadjuvant therapy were excluded to obtain a more homogeneous study population and to avoid potential confounding effects of preoperative systemic or radiation treatment on postoperative complications, surgical complexity, and survival outcomes.

Before treatment allocation, all patients were discussed in a multidisciplinary tumor board consisting of thoracic surgeons, pulmonologists, medical oncologists, radiation oncologists, and radiologists. Treatment recommendations were based on tumor stage, imaging findings, comorbidities, pulmonary function, and overall operability. During the study period, a dedicated geriatrician was not routinely involved in the multidisciplinary decision-making process.

### 2.2. Data Assessments and Sources

Patient demographics, perioperative variables, and oncological data were retrieved from a retrospectively maintained institutional database. Tumor stage was assigned according to the 8th edition of the TNM classification system [19], while histological tumor subtypes were categorized in accordance with the World Health Organization (WHO) classification of thoracic tumors [20].

All anatomical lung resections were performed either through a video-assisted thoracoscopic surgery (VATS) approach or by open thoracotomy. In the present study, minimally invasive surgery refers exclusively to VATS. The terms “VATS” and “minimally invasive approach or surgery” are therefore used interchangeably throughout the manuscript.

Collected variables comprised demographic and clinical characteristics including age, sex, body mass index (BMI), smoking status, and comorbidities. Laboratory parameters obtained within 1 to 5 days before surgery included complete blood count, C-reactive protein (CRP), creatinine, lactate dehydrogenase (LDH), and serum albumin levels. Preoperative pulmonary function assessment consisted of functional vital capacity (FVC), forced expiratory volume in one second (FEV1), diffusion capacity of the lung for carbon monoxide (DLCO SB), and the FEV1/FVC ratio. Tumor stage was initially evaluated according to clinical and radiological findings (cTNM), and detailed histopathological characteristics were recorded. Surgical outcomes were assessed using postoperative morbidity, 30-day mortality, and length of hospital stay. Perioperative blood transfusion was defined as the administration of allogenic packed red blood cells either intraoperatively or during the postoperative hospitalization period. Transfusion data were obtained from anesthesiology records, intensive care unit (ICU) documentation, and the institutional electronic medical record system. Perioperative blood transfusions were administered either intraoperatively in cases of significant blood loss or postoperatively according to clinical indication. In the postoperative setting, red blood cell transfusion was generally considered when hemoglobin levels fell below 8 g/dL, considering the patient’s clinical status and hemodynamic condition. Prolonged air leak (PAL) was defined as the presence of an air leak persisting for more than 5 postoperative days following lung resection.

The Clavien-Dindo classification and its adaption for thoracic surgery, the thoracic morbidity and mortality (TMM) classification system, were used to grade surgical complications [18,21]. Major surgical complications were defined as complications ≥ grade 3.

Postoperative follow-up was conducted through thoracic surgical, oncological, and pulmonological outpatient clinics. Patients who continued their care outside our institution were followed by community-based oncologists, pulmonologists, or general practitioners. To ensure complete follow-up, survival status and clinical outcome data were verified through review of medical records and, when necessary, direct telephone contact with patients, relatives, or treating physicians. Survival data were collected from the date of surgery until death or the last follow-up, which was completed in February 2024.

### 2.3. Outcomes

The present study aimed to evaluate perioperative outcomes following major lung cancer resection in patients aged 80 years and older and to identify factors associated with an increased risk of major postoperative complications and mortality. To achieve this objective, demographic characteristics, comorbidities, laboratory parameters, pulmonary function measurements, and radiological and histopathological findings were systematically analyzed. Associations between these variables and postoperative outcomes were assessed using the Thoracic Morbidity and Mortality (TMM) classification, comparing patients with no or minor complications (TMM 0–2) to those who developed major complications (TMM ≥ 3) [18].

Finally, overall survival of the lung cancer patients upon major surgical resection was analyzed as well.

### 2.4. Data Analysis

All statistical analyses were conducted using R software (version 4.2.2). Continuous variables were assessed for distributional characteristics and are presented as mean ± standard deviation or median with interquartile range, as appropriate. Group comparisons were performed using the unpaired Student’s *t*-test for normally distributed variables and the Mann–Whitney U test for non-normally distributed data. Categorical variables were compared using the Chi-square test or Fisher’s exact test when expected cell counts were low. Data visualization was generated using the ggplot and ggpubr packages. Associations with major postoperative complications were explored through univariable and multivariable logistic regression analyses using the base R “glm()” function. Forest plots were created with the SjPlot package [22]. Variables considered for multivariable logistic regression were selected based on a combination of statistical significance in univariable analyses (*p* < 0.05), clinical relevance, and assessment of collinearity. When multiple variables represented closely related clinical domains, one representative parameter was selected to reduce multicollinearity and improve model stability. Given the limited number of major complication events, the multivariable analysis was considered exploratory and intended primarily to identify potential independent associations rather than establish definitive causal relationships. Overall survival was estimated using the Kaplan–Meier method and compared between groups with the log-rank test. Variables identified as significant in the univariable analyses were subsequently entered into Cox proportional hazards regression models to evaluate their independent association with survival outcomes. Overall survival was calculated from the date of lung cancer resection to the date of death from any cause or the last available follow-up for censored patients. *p*-values < 0.05 were considered statistically significant.

To evaluate the robustness of the observed association between perioperative blood transfusion and overall survival in the presence of a limited sample size and potential residual confounding, a non-parametric bootstrap validation procedure was performed using the boot package in R. A total of 100,000 bootstrap samples were generated by resampling with replacement from the original cohort. Within each bootstrap sample, a multivariable Cox proportional hazards model was fitted to estimate the adjusted hazard ratio for perioperative blood transfusion. The model included clinically relevant covariates, including histological subtype, tumor staging, and tumor size. The resulting bootstrap distribution was used to assess the stability and uncertainty of the estimated hazard ratio.

## 3. Results

### 3.1. Cohort Characteristics

Between January 2014 and December 2023, 88 patients aged ≥80 years with pathologically proven lung cancer types, undergoing anatomic lung resection were retrospectively included. The analyzed cohort (42% female patients) included patients with a median age of 82.3 (±2.3) years and a median BMI of 25.75 (±5.6) kg/m^2^. Using the standardized TMM classification, patients were categorized into two groups according to the severity of postoperative complications: Minor or no complications (TMM 0–2, 64 patients, 72.7%) and major complications (TMM ≥ 3, 24 patients, 27.3%), respectively.

There was no statistically significant age difference between the groups (82.3 (±1.9) years vs. 82.6 (±3.2) years, *p* = 0.659). Patients with major complications were admitted with significantly lower lung function parameters in comparison to the TMM 0–2 patients (FEV1: 79.2 (±16.9) vs. 89.1 (±18.7) % predicted, *p* = 0.0308; DLCO SB: 56.9 (±12.2) vs. 68.8 (±17.7) % predicted, *p* = 0.0019). No significant differences in the distribution of patients’ comorbidities were observed between groups. The patients’ demographics are summarized in Table 1.

No significant differences regarding tumor histology, side, lobar distribution, and lymph node involvement were reported between groups. In the major complication group, the tumor size in CT findings as well as in pathological findings was significantly larger than in the TMM 0–2 group (4.89 cm (±2.26) vs. 3.34 cm (±1.75), *p* = 0.0004531 and 5.21 cm (±2.34) vs. 3.46 cm (±1.85), *p* = 0.0009684, Table 2).

The characteristics of the surgical procedures and postoperative outcomes are presented in Table 3.

No significant differences on the extent of tumor resection were noted (*p* = 0.3592). However, the TMM ≥ 3 group included significantly more patients undergoing open surgery by thoracotomy (70.8% vs. 29.6%, *p* = 0.00065).

Additionally, the TMM ≥ 3 group exhibited a significantly prolonged hospital stays (17 days vs. 10 days, *p* = 0.00053), extended ICU stay (2 days vs. 1 day, *p* = 0.000003), and higher 30-day postoperative mortality (5 (20.8%) vs. 0 (0%), *p* = 0.001), respectively.

The duration of drainage and the duration of surgery were increased in the TMM ≥ 3 group, suggesting a potential association; however, these differences were not statistically significant (*p* = 0.07 and *p* = 0.058), although a trend was observed. Perioperatively, patients in TMM ≥ 3 group received significantly more blood transfusions (54.1% vs. 6.2%, *p* < 0.00001).

In the TMM ≥ 3 group, preoperative laboratory findings showed decreased hemoglobin and increased CRP levels, pointing toward a possible association with major complications; however, statistical significance was not reached (*p* = 0.06 and *p* = 0.08). In contrast, lower levels of albumin were significantly associated with the occurrence of major complications (3.65 (±0.668) vs. 3.92 (±0.505), *p* = 0.023). The laboratory parameters in both groups are presented in Table 4.

Overall, 38 patients developed a total of 110 postoperative complication events. 15 patients in TMM ≥ 3, and two in TMM 0–2 groups experienced more than one postoperative complication, respectively. The most frequently observed complications in the TMM ≥ 3 group were respiratory failure (25.0%), pneumonia (20.8%), pleural effusion (20.8%), pneumothorax (16.6%), atrial flutter (12.5%), delirium (8.3%), hemothorax (8.3%), pulmonary embolism (8.3%), and prolonged air leak (PAL) (8.3%). Two patients in the TMM ≥ 3 group developed PAL. In one patient, postoperative hemothorax required surgical revision on the day of surgery, followed by a persistent air leak lasting 20 days. In the second patient, persistent postoperative air leakage necessitated surgical revision on postoperative day 6, during which a ruptured pulmonary bulla was identified and repaired. The distribution and frequency of specific complications are summarized in Table 5.

Following review of the final pathological findings in the multidisciplinary tumor board, adjuvant treatment was recommended in 19 patients according to current oncological guidelines and individual clinical characteristics.

### 3.2. Logistic Regression Analysis of Risk Factors

**Figure 1:**
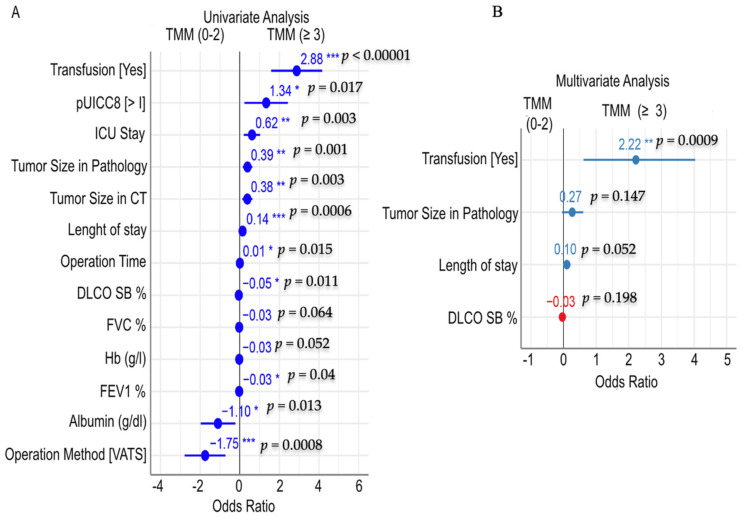
Univariable and multivariable logistic regression model predicting postoperative major complications in older patients undergoing major surgical lung resection. (**A**)—Forest plot showing odds ratio with 95% confidence intervals from the univariable logistic regression analysis. (**B**)—Forest plot showing multivariable regression analysis. *p*-values < 0.05 were considered significant. Abbreviations: TMM: Thoracic Morbidity and Mortality; p: Pathological staging based on surgical resection; UICC: Union for International Cancer Control; ICU: Intermediate Care Unit; CT: Computed Tomography; DLCO SB: Diffusion capacity of the lung for carbon monoxide in single breath; FVC: functional vital capacity; Hb: Hemoglobin; FEV1: Forced expiratory volume in one second; VATS: Video-assisted thoracoscopic surgery.

In the univariable analysis of the entire cohort, lower levels of serum albumin were associated with major postoperative complications (*p* = 0.013). Additional variables associated with major complications were perioperative blood transfusions (*p* < 0.00001), pathological tumor staging (pUICC8 > 1, *p* = 0.017), CT-measured tumor size (cT, *p* = 0.003) and pathological tumor size (pT, *p* = 0.001). In terms of pulmonary function, reduced values of DLCO SB and FEV1 were associated with major postoperative complications (*p* = 0.011 and *p* = 0.03). Moreover, longer duration of surgery was associated with major complications (*p* = 0.015), whereas VATS was related to a lower risk of complications than open surgery (*p* = 0.0008). Patients experiencing major complications showed a significantly prolonged ICU and overall hospital stay (*p* = 0.003 and *p* = 0.0006) (Figure 1A).

To explore variables independently associated with major postoperative complications, a multivariable logistic regression analysis was performed. In multivariable analysis, perioperative blood transfusion remained independently associated with occurrence of major postoperative complications (OR = 2.21, CI (0.67–3.92), *p* = 0.0009). The logistic regression model is summarized in Figure 1B.

### 3.3. Survival Analysis

**Figure 2:**
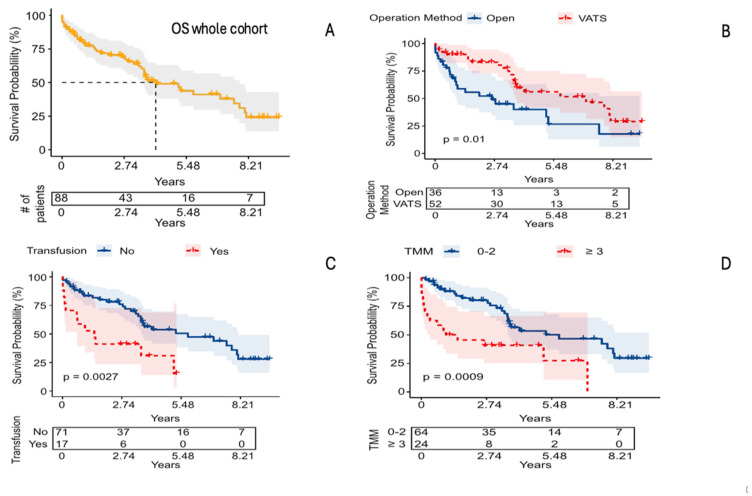
Kaplan–Meier survival analysis including patients at risk, reported events (deaths) and patients censored (February 2024) addressing the whole cohort (**A**), surgical approach (**B**), perioperative transfusion (**C**), postoperative no or minor complications and postoperative major complications (**D**). Comparison of the survival estimates was analyzed by log-rank test. *p*-values < 0.05 were considered significant. Abbreviations: VATS: Video-assisted thoracoscopic surgery; TMM: Thoracic Morbidity and Mortality; The symbol ‘#‘ indicates the number of patients.

Figure 2A shows that the median overall survival for all patients was 49 months. The 1-, 3-, and 5-year survival rates were 77.4%, 65.6%, and 49.1%, respectively. During the follow-up period, a total of 44 deaths were observed.

Survival outcomes were associated with the surgical approach.

As shown in Figure 2B, patients undergoing VATS lung resection demonstrated improved survival compared with those undergoing open lung resection (log-rank *p* = 0.01). In univariable analysis, VATS was associated with a significantly reduced risk of mortality compared to open surgery (HR 0.47, 95% CI 0.26–0.85, *p* = 0.013). After 2.74 years of follow-up, 57.7% (30/52) of patients in the VATS group were alive, compared with 36.1% (13/36) in the open surgery group. This difference remained evident after 5.48 years (13 vs. 3 patients) and 8.21 years (9.6% (5/52) vs. 5.6% (2/36)).

Patients receiving perioperative blood transfusion showed significantly reduced overall survival compared to those without transfusion (log-rank *p* = 0.0027). In univariable Cox regression analysis, blood transfusion was associated with an increased risk of mortality (HR 2.72, 95% CI 1.37–5.42, *p* = 0.004), (Figure 2C).

In Figure 2D a Kaplan–Meier analysis demonstrated significantly reduced overall survival in patients with major complications (TMM ≥ 3) compared to those with minor or no complications (TMM 0–2) (log-rank *p* = 0.0009).

Albumin levels were significantly associated with overall survival, with higher albumin levels (>3.5 g/dL) being protective (*p* = 0.002).

In multivariable Cox regression analysis, major complications (TMM ≥ 3) remained the only independent predictor of reduced overall survival (HR 1.85, 95% CI 1.33–2.58, *p* < 0.001). Perioperative blood transfusion showed a strong association with mortality; however, this did not reach statistical significance after adjustment (HR 2.81, 95% CI 0.96–8.17, *p* = 0.058). Given the borderline statistical significance of perioperative blood transfusion in the multivariable Cox regression model, a bootstrap-based sensitivity analysis was performed to assess the robustness of this finding and the potential influence of sampling variability. A total of 100,000 bootstrap samples were generated with replacement from the original cohort. Across the bootstrap samples, the hazard ratio for perioperative blood transfusion was 2.18 (95% bootstrap CI 0.78–6.28, *p* = 0.138). Although the direction and magnitude of the association remained consistent with the primary analysis, statistical significance was not retained. These findings suggest that the observed association may be sensitive to sampling variability and should therefore be interpreted with caution. Further validation in larger cohorts is warranted.

Surgical approach and perioperative albumin levels were not independently associated with overall survival, Table 6.

## 4. Discussion

As the global population ages, the number of octogenarians with lung cancer rises quickly [2,23]. Surgical resection remains the standard treatment for medically operable patients with early-stage NSCLC. However, in octogenarian patients, the potential benefits of surgery must be carefully balanced against operative risks [24]. Alternative treatment approaches, including stereotactic body radiotherapy (SBRT), have demonstrated promising oncological outcomes in selected patients and should be considered within a multidisciplinary treatment framework [6]. This study included 88 old patients undergoing anatomical lung resection. A standardized TMM grading system [18] was used to evaluate the severity of postoperative complications. The aim of the present study was to explore potential independent risk factors associated with major postoperative complications and long-term survival in patients over 80 years undergoing anatomical lung resection.

In our analysis, 27.3% of patients encountered major complications (TMM ≥ 3), a percentage that aligns with prior research on older lung cancer patients [25]. Notably, our survival study showed that patients with TMM ≥ 3 complications had a much poorer overall survival rate than those with no or minor complications (*p* = 0.0009). This corroborates the findings of Salati et al., indicating that the TMM method effectively detects the influence of postoperative complications on patient prognosis—a differentiation that is not easily discernible by conventional ESTS-defined complication classifications [26]. This study further emphasizes the clinical significance of a graded assessment system for aged patients, offering a benchmark for future perioperative categorization and tailored treatment approaches.

Our univariable logistic regression analysis further substantiated that reduced preoperative DLCO-SB functions as a risk factor for major complications. Pulmonary diffusing capacity (DLCO) represents a measure of the alveolar-capillary membrane exchange capacity. It is considered a more reliable indicator of pulmonary function than FEV1% alone [27]. Our study eventually demonstrated that patients in the TMM ≥ 3 group displayed significantly decreased DLCO-SB values in comparison to those in the TMM 0–2 group (*p* = 0.0019). This result corroborates the findings of a comprehensive cohort research conducted by Ferguson et al., which revealed that diminished DLCO values serve as an independent predictor of postoperative problems, even among patients exhibiting normal FEV1 [28]. Moreover, an additional investigation indicated that DLCO not only forecasts short-term complications but is also significantly correlated with the long-term course [29].

Perioperative blood transfusion constitutes an additional high-risk factor for severe postoperative complications in older lung cancer patients, distinct from DLCO. Our results support this observation, demonstrating significantly reduced overall survival in patients who received transfusions compared to those who did not (*p* = 0.0027). Notably, the transfusion rate was markedly higher in the TMM ≥ 3 group (54.1%) compared to the TMM 0–2 group (6.2%). By comparison, in our previously published cohort, the overall perioperative blood transfusion rate was 11.3%, which was substantially lower than the rate observed in the present study (19.3%, 17/88 patients). This difference may reflect the higher proportion of complex resections and the advanced age of the current study population. In that study, preoperative anemia, thoracotomy, multilobar resections, and tumor burden were identified as key predictors of transfusion requirement, emphasizing the multifactorial nature of perioperative transfusion risk [30]. The association between perioperative blood transfusion and adverse postoperative outcomes should be interpreted with caution. Reverse causality cannot be excluded, as patients requiring transfusion may have experienced greater intraoperative blood loss, technically more complex procedures, or perioperative complications that themselves contributed to worse outcomes. Therefore, blood transfusion may represent both a marker of operative complexity and a potential contributor to postoperative morbidity and mortality. The mechanisms underlying the adverse impact of transfusion are complex. First, transfusion may lead to transfusion-related immune modulation (TRIM), which can compromise immune function, thereby reducing resistance to infections and potentially promoting tumor recurrence [31]. Cho et al.’s meta-analysis showed that perioperative transfusion significantly reduced disease-free survival and overall survival in lung cancer patients, especially in those with adenocarcinoma [32]. Secondly, blood transfusion has been linked to severe adverse events, including transfusion-related acute lung injury (TRALI) and thrombosis, which may contribute to an increased risk of postoperative mortality [33]. In a study by Thomas et al. on lung resection, blood transfusion was identified as the strongest predictor of 30-day mortality (OR = 10) and was also associated with respiratory failure and infectious complications [34]. Considering these findings, the implementation of blood-conserving strategies is essential when treating old or very old patients undergoing lung cancer surgery. These encompass preoperative anemia rectification, enhanced surgical methodologies, and the formulation of transfusion thresholds.

From an oncological point of view, tumor size may serve as a prognostic indicator to a certain degree, a conclusion corroborated by our investigation. Zhang et al. showed that tumor size is an independent predictor of postoperative survival in patients with stage IIIA-N2 lung cancer. Patients with tumors that are 2 cm or smaller have far better results than those with larger tumors [35]. In older individuals, bigger tumors frequently make surgery harder, lower the body’s ability to heal, and raise the danger of bleeding, which makes postoperative adverse events more likely. Consequently, in devising treatment plans for this cohort of patients, thorough attention must be paid to individual variances to create customized surgical strategies and care protocols in the postoperative period. Advanced pathological stage may represent an upstream factor linking larger tumor burden, greater operative complexity, perioperative transfusion requirements, postoperative complications, and reduced long-term survival. In our cohort, patients with major complications more frequently presented with Stage III disease and larger tumors. However, the presence of long-term survivors even among patients with major complications suggests that survival outcomes remain influenced by multiple factors beyond pathologic stage alone, including performance status, perioperative course, and individual tumor characteristics. The prognostic relevance of tumor size should also be interpreted in the context of evolving perioperative treatment strategies. Recent trials such as KEYNOTE-671 have demonstrated promising results with multimodal treatment concepts and did not specifically exclude older patients [36]. Consequently, future studies evaluating outcomes in octogenarians should consider the potential impact of contemporary perioperative therapies on survival.

Presently, video-assisted thoracoscopic surgery (VATS) has replaced conventional thoracotomy in approximately two thirds of resectable lung cancer cases due to its advantages of reduced surgical trauma and lower rates of postoperative complications, including pain, impaired pulmonary function, and delayed wound healing [37]. In our cohort, older patients undergoing VATS demonstrated significantly improved overall survival compared with those undergoing open surgery (*p* = 0.01), with the VATS cohort also presenting a reduced complication rate (29.2% vs. 70.8%). Our findings are largely consistent with those reported by Port et al. in a study of 121 patients aged ≥80 years [38]. Importantly, recent high-level evidence further supports the advantages of minimally invasive surgery. Harris et al. performed an individual patient data meta-analysis of randomized controlled trials including 1185 patients undergoing lobectomy for early-stage NSCLC and demonstrated that VATS was associated with a 21% reduction in mortality risk compared with open surgery (HR 0.79, 95% CI 0.65–0.96), while maintaining equivalent disease-free survival [39]. These findings suggest that the benefits of VATS extend beyond improved perioperative recovery and may also translate into superior long-term survival. Our observations are consistent with these results, as patients undergoing VATS in our cohort experienced both fewer major postoperative complications and significantly improved overall survival. Minimally invasive approaches are of particular importance in older patients, given their reduced physiological reserve frequently due to deterioration of the cardiopulmonary function and others like the loss of muscle-mass compromising the respiratory performance. Nevertheless, the successful implementation of VATS depends heavily on surgical expertise and technical proficiency. Inadequate patient selection or intraoperative technical challenges may require conversion to open surgery, potentially increasing perioperative risk [40]. Overall, patients in the VATS group exhibited lower complication rates (35.0% vs. 63.0%), shorter hospital stay, and reduced ICU admission rates after major surgery. The relatively long drainage duration observed in both groups should be interpreted in the context of the institutional chest tube management strategy applied during the study period. Chest tubes were generally removed only when pleural drainage volume was below 200 mL within 24 h. Furthermore, several patients experienced prolonged or intermittent postoperative air leaks. Despite the lack of a standardized definition, prolonged air leak is generally regarded as the persistence of an air leak for more than 5 to 7 days after lung surgery [41]. Previous studies have demonstrated that PAL is associated with increased postoperative morbidity, prolonged hospitalization, and higher healthcare utilization [42]. In our cohort, two patients in the TMM ≥ 3 group developed PAL. Although the overall incidence was low, these cases substantially contributed to extended drainage duration and postoperative recovery. Therefore, PAL should be recognized as an important determinant of postoperative outcome in octogenarian patients undergoing major lung resection and may partly explain the prolonged drainage duration observed in the present study.

Our findings indicate that reduced serum albumin levels are associated with both an increased risk of major postoperative complications and decreased overall survival in elderly patients undergoing lung cancer surgery. Hypoalbuminemia, frequently reflecting underlying malnutrition, is linked to multiple physiological impairments that may predispose patients to postoperative complications. Supporting this, Lee et al. demonstrated that grade ≥1 hypoproteinemia was significantly associated with post-chemotherapy pulmonary complications in patients with locally advanced NSCLC [43]. Furthermore, Morelli et al. reported that hypoalbuminemia was associated with reduced overall survival, reinforcing its prognostic relevance [44]. However, the exact pathophysiological mechanisms underlying this association remain incompletely understood. A review by Kim et al. did not establish a direct causal relationship between low serum albumin levels and adverse outcomes [45], suggesting that hypoalbuminemia may primarily reflect underlying patient frailty rather than acting as a direct contributor. Accordingly, interventions aimed solely at correcting perioperative hypoalbuminemia, such as intravenous albumin administration, appear to have limited impact on clinical outcomes. From a clinical perspective, low serum albumin at admission should therefore be considered primarily as a prognostic marker, aiding in risk stratification and perioperative decision-making.

Our study showed that elderly patients had a median overall survival of 49 months, with corresponding 1-, 3-, and 5-year survival rates of 77.4%, 65.6%, and 49.1%, respectively. These findings are consistent with several previous investigations [46,47]. Remarkably, 50% of patients survived beyond 5 years, with seven patients (7.9%) living longer than 8 years. This underscores that carefully selected elderly patients undergoing anatomical lung resection can achieve meaningful long-term survival. This is consistent with recent prospective data demonstrating comparable long-term survival and quality of life between carefully selected patients aged ≥80 years and younger individuals undergoing surgery for early-stage lung cancer [5].

In the last ten years, better perioperative care and technical improvements have lowered the mortality rate for lung resection in people over 80 from 8–15% to the present 2–4% range [48,49,50,51]. In this study, the 30-day postoperative mortality rate was merely 1.9%, akin to that of younger patient cohorts, indicating the advancements in modern thoracic surgical methods. Consequently, age should not be regarded as an unequivocal contraindication for surgery; the essential factor is thorough preoperative evaluation and risk stratification [14,52].

The clinical value of this study resides in its provision of evidence-based support for tailored treatment of old and very old (≥80 years) lung cancer patients. Our study revealed critical risk factors, including diminished DLCO, perioperative blood transfusion, and surgical technique, facilitating more accurate preoperative risk classification and effectively mitigating severe postoperative consequences. For patients who are at high risk, we should: 1. Improve preoperative care by adding pulmonary rehabilitation training, nutritional assistance, and fixing anemia; 2. Improve surgical methods by putting VATS procedures first and lowering the need for blood transfusions; 3. Improve perioperative surveillance to proactively detect and address problems. For patients with significantly diminished DLCO, it is imperative to prioritize more conservative interventions such as sub lobar resection, or to explore alternative therapies like stereotactic body radiation therapy (SBRT) following a multidisciplinary team deliberation [53]. In recent years, SBRT has shown good local control rates and minimal complication rates in older lung cancer patients. This gives new hope to people who are at high risk for surgery after an evaluation [54]. Nonetheless, there is a deficiency of high-quality randomized controlled trials comparing the efficacy of surgery against SBRT in old and very old patients, indicating a vital avenue for future research.

Limitations: Several limitations with this study should be acknowledged. First, it is a retrospective study conducted at a single center, characterized by a limited sample size and a significant level of selection bias. In addition, due to retrospective study design we were unable to determine the total number of octogenarian patients evaluated for surgical treatment during the study period who were ultimately not offered resection. Consequently, the extent of potential selection bias cannot be fully assessed. It is likely that patients selected for surgery represented a relatively fit subgroup with favorable functional status and operability, which may limit the generalizability of the present findings to the broader population of older patients with lung cancer. Third, the relatively small number of major postoperative complication events restricted the complexity of multivariable models that could be reliably supported. Although variable selection was based on clinical relevance and assessment of collinearity, the stability of the multivariable estimates may have been limited, and the possibility of model overfitting cannot be completely excluded. Consequently, the findings of the multivariable analyses should be interpreted as exploratory and hypothesis-generating rather than confirmatory and require validation in larger prospective cohorts. Furthermore, it does not include a comprehensive review of geriatric assessment markers such preoperative frailty scores and sarcopenia, which could have a big effect on prognosis. Fourth, it does not examine the varying effects of distinct complication types (e.g., cardiovascular, respiratory) on survival. Future research should utilize multicenter, large-sample prospective designs and incorporate more extensive geriatric assessment instruments to enhance treatment methods for this at-risk population.

## 5. Conclusions

The standardized TMM grading system accurately evaluates the severity of surgical complications in elderly lung cancer patients. In this cohort of octogenarian patients undergoing anatomical lung resection, major postoperative complications were strongly associated with impaired long-term survival. VATS was associated with fewer major complications and improved survival outcomes, consistent with emerging evidence from randomized studies supporting minimally invasive approaches in lung cancer surgery [39]. Perioperative blood transfusion was associated with adverse postoperative and survival outcomes; however, this finding should be interpreted with caution given the retrospective study design and the potential influence of residual confounding and reverse causality. Further prospective multicenter studies are required to validate these findings and to define optimal perioperative management strategies in this high-risk population.

## Figures and Tables

**Table 1:** Demographics of patients undergoing lung cancer resection classified by TMM groups.

Variables	TMM: 0–2 *n* = 64	TMM: ≥3 *n* = 24	*p*-Value
**Age, years,** (Mean ± SD)	82.3 (±1.99)	82.6 (±3.20)	0.6597
**Sex**			1.000
Male, *n* (%)	37 (57.8)	14 (58.3)	
Female, *n* (%)	27 (42.2)	10 (41.7)	
**BMI [kq/m^2^]**			0.8107
≥25	34 (53.1)	14 (58.3)	
<25	30 (46.9)	10 (41.7)	
**Alcohol and Tobacco use *n* (%)**	42 (65.6)	16 (66.6)	1.000
Smoking status [Ever smoker], *n* (%)	38 (59.3)	15 (62.5)	1.000
Alcohol consumption [Yes], *n* (%)	16 (25)	3 (12.5)	0.255
**ASA**			0.846
2, *n* (%)	14 (21.8)	4 (16.6)	
3, *n* (%)	46 (71.8)	18 (75)	
4, *n* (%)	4 (6.2)	2 (8.3)	
**ECOG PS**			0.0566
0, *n* (%)	55 (85.9)	16 (66.6)	
1, *n* (%)	8 (12.5)	8 (33.3)	
2, *n* (%)	1 (1.5)	0 (0)	
**Lung function parameters**			
FEV1 (predicted, %), (Mean ± SD)	89.1 (±18.7)	79.2 (±16.9)	0.0308
DLCO SB (predicted, %), (Mean ± SD)	68.8 (±17.7)	56.9 (±12.2)	0.0019
FVC (predicted, %), (Mean ± SD)	90.2 (±16.1)	82.1 (±16.5)	0.0834
FEV1/FVC (predicted, %), (Mean ± SD)	96.9 (±10.7)	96.5(±13.5)	0.5235
**Comorbidities**			
Respiratory, *n* (%)	11 (17.1)	5 (20.8)	0.7589
Cardiac, *n* (%)	26 (40.6)	12 (50)	0.4748
Renal, *n* (%)	4 (6.2)	2 (8.3)	0.6659
Liver, *n* (%)	4 (6.2)	0 (0)	0.5712
Diabetes mellitus, *n* (%)	10 (15.6)	6 (25)	0.357
**CCI**			0.1819
6	30 (46.8)	10 (41.6)	
7	22 (34.3)	5 (20.8)	
8	8 (12.5)	7 (29.1)	
9	2 (3.1)	0 (0)	
10	2 (3.1)	1 (4.1)	
11	0 (0)	1 (4.1)	

For continuous variables, a non-parametric Mann–Whitney U test was performed. For binary variables, the Pearson Chi-square test or Fisher’s exact test was performed. *p*-values < 0.05 are statistically significant. Abbreviations: TMM: Thoracic Morbidity and Mortality; SD: Standard Deviation; BMI: Body mass index; ASA: American society of anesthesiologists; ECOG PS: Eastern cooperative oncology group performance status; FEV1: Forced expiratory volume in one second; FVC: functional vital capacity; DLCO SB: Diffusion capacity of the lung for carbon monoxide in single breath; CCI: Charlson Comorbidity Index.

**Table 2:** Tumor characteristics and surgical aspects of patients undergoing surgical resection classified by TMM groups.

Variables	TMM: 0–2 *n* = 64	TMM: ≥3 *n* = 24	*p*-Value
**Tumor histology**			0.3403
Adenocarcinoma, *n* (%)	41 (64)	13 (54.1)	
Squamous cell carcinoma, *n* (%)	14 (21.8)	8 (33.3)	
Adenosquamous carcinoma, *n* (%)	1 (1.5)	0 (0)	
NOS carcinoma, *n* (%)	2 (3.1)	0 (0)	
Typical carcinoid, *n* (%)	6 (9.3)	1 (4.1)	
Atypical carcinoid, *n* (%)	0 (0)	1 (4.1)	
Large-cell carcinoma, *n* (%)	0 (0)	1 (4.1)	
**Tumor side**			1.000
Right, *n* (%)	38 (59.3)	15 (62.5)	
Left, *n* (%)	26 (40.7)	9 (37.5)	
**Tumor localization**			0.05136
LLL, *n* (%)	12 (18.7)	1 (4.1)	
LMB, *n* (%)	0 (0)	1 (4.1)	
LUL, *n* (%)	14 (21.8)	7 (29.1)	
RLL, *n* (%)	11 (17.1)	9 (37.5)	
RML, *n* (%)	5 (7.8)	0 (0)	
RUL, *n* (%)	22 (34.3)	6 (25)	
**pUICC, 8th Edition**			0.02814
I, *n* (%)	32 (50)	5 (20.8)	
II, *n* (%)	18 (28.1)	8 (33.3)	
III, *n* (%)	14 (21.8)	11 (45.8)	
**pT stage**			0.04679
1, *n* (%)	26 (40.6)	3 (12.5)	
2, *n* (%)	20 (31.25)	6 (25)	
3, *n* (%)	12 (18.75)	9 (37.5)	
4, *n* (%)	5 (7.8)	6 (25)	
**pN stage**			0.2515
0, *n* (%)	48 (75)	14 (58.3)	
1, *n* (%)	8 (12.5)	4 (16.6)	
2, *n* (%)	7 (10.9)	4 (16.6)	
Unknown, *n* (%)	1 (1.5)	2 (8.3)	
**Tumor size in CT** in cm, (Mean ± SD)	3.34 (±1.75)	4.89 (±2.26)	0.0004531
**Tumor size in pathology** in cm, (Mean ± SD)	3.46 (±1.85)	5.21 (±2.34)	0.0009684
**Pleura visceralis infiltration**, *n* (%)	26 (40.6)	9 (37.5)	1.000

For continuous variables, a non-parametric Mann–Whitney U test was performed. For binary variables, the Pearson Chi-square test or Fisher’s exact test was performed. *p*-values < 0.05 are statistically significant. Abbreviations: NOS: Not otherwise specified; LLL: Left lower lobe; LMB: Left main bronchus; LUL: Left upper lobe; RLL: Right lower lobe; RML: Right middle lobe; RUL: Right upper lobe; p: Pathological staging based on surgical resection; UICC: Union for International Cancer Control.

**Table 3:** Surgical Characteristics and Postoperative Outcomes Stratified by TMM Classification.

Variables	TMM: 0–2 *n* = 64	TMM: ≥3 *n* = 24	*p*-Value
Surgical approach			0.0006549
Thoracotomy, *n* (%)	19 (29.6)	17 (70.8)	
Minimally invasive, *n* (%)	45 (70.4)	7 (29.2)	
Surgical resection			0.3592
Segmentectomy, *n* (%)	5 (7.8)	1 (4.1)	
Lobectomy, *n* (%)	54 (84.3)	19 (79.1)	
Bilobectomy, *n* (%)	5 (7.8)	3 (12.5)	
Pneumonectomy, *n* (%)	0 (0)	1 (4.1)	
Surgery time (min), (Median, IQR)	155 (124.7–206)	187 (142.5–269.7)	0.05839
ICU stay, days, (Median, IQR)	1 (1–1)	2(1–4)	0.000003
LOS, days, (Median, IQR)	10 (8–14)	17 (14–24.25)	0.0005325
Duration of drainage, days, (Median, IQR)	7 (5–11)	10.5 (6–15)	0.0739
Readmission in 30 days, *n* (%)	8 (12.5)	2 (8.3)	0.7214
Mortality in 30 days, *n* (%)	0 (0)	5 (20.8)	0.001085
Transfusion [Yes], *n* (%)	4 (6.2)	13 (54.1)	0.0000002

For continuous variables, a non-parametric Mann–Whitney U test was performed. For binary variables, the Pearson Chi-square test or Fisher’s exact test was performed. *p*-values < 0.05 are statistically significant. Abbreviations: ICU: Intermediate care unit; LOS: Length of hospital stay; IQR: Interquartile range.

**Table 4:** Preoperative laboratory parameters of patients undergoing surgical resection classified by TMM groups.

Variables	TMM: 0–2 *n* = 64	TMM: ≥3 *n* = 24	*p*-Value
Leucocytes (/nL, Mean ± SD)	7.71 (±2.14)	9.22 (±4.19)	0.1786
Hb (g/L, Mean ± SD)	128 (±16.8)	120 (±19.2)	0.06726
Erythrocytes (/pL, Mean ± SD)	4.25 (±0.536)	4.08 (±0.639)	0.2729
Thrombocytes (/nL, Mean ± SD)	247 (±82.8)	275 (±124)	0.4203
CRP (mg/dL, Mean ± SD)	1.25 (±3.59)	3.76 (±7.04)	0.08616
Creatinin (mg/dL, Mean ± SD)	0.995 (±0.252)	0.958(±0.282)	0.5041
Albumin (g/dL, Mean ± SD)	3.92 (±0.505)	3.65 (±0.668)	0.02318
LDH (U/L, Mean ± SD)	193 (±47.9)	206 (±43.6)	0.1266

For continuous variables, a non-parametric Mann–Whitney U test was performed. For binary variables, the Pearson Chi-square test or Fisher`s exact test was performed. *p*-values < 0.05 are statistically significant. Abbreviations: Hb: Hemoglobin; CRP: C-reactive protein; LDH: Lactate dehydrogenase.

**Table 5:** Distribution and frequency of postoperative complications in the study cohort.

Complications	TMM: 0–2 *n* = 64	TMM: ≥3 *n* = 24
Pneumonia, *n* (%)	9 (14.1)	5 (20.8)
Respiratory failure, *n* (%)	0 (0)	6 (25)
Myocardial infarction, *n* (%)	0 (0)	1 (4.1)
Atrial flutter, *n* (%)	3 (4.6)	3 (12.5)
PAL, *n* (%)	1 (1.5)	2 (8.3)
Pneumothorax, *n* (%)	0 (0)	4 (16.6)
Renal failure, *n* (%)	1 (1.5)	1 (4.1)
Middle lobe syndrome, *n* (%)	0 (0)	1 (4.1)
Recurrent nerve palsy, *n* (%)	2 (3.1)	1 (4.1)
Phrenic nerve palsy, *n* (%)	0 (0)	1 (4.1)
Delirium, *n* (%)	0 (0)	2 (8.3)
Dysphagia, *n* (%)	0 (0)	1 (4.1)
Thoracic wall herniation, *n* (%)	0 (0)	1 (4.1)
Hemothorax, *n* (%)	0 (0)	2 (8.3)
Pulmonary edema, *n* (%)	0 (0)	1 (4.1)
Pleural effusion, *n* (%)	0 (0)	5 (20.8)
Hollow organ perforation, *n* (%)	0 (0)	1 (4.1)
GI bleeding, *n* (%)	0 (0)	1 (4.1)
Pulmonary embolism, *n* (%)	0 (0)	2 (8.3)
Death, *n* (%)	0 (0)	5 (20.8)

Abbreviations: PAL: Prolonged air leak; GI: Gastrointestinal.

**Table 6:** Multivariable Cox Regression Analysis of Factors Associated with Overall Survival.

Variables	HR	95% CI	*p*-Value
TMM ≥ 3	1.85	1.33–2.58	<0.001
Surgical approach	1.09	0.47–2.52	0.826
Albumin	0.60	0.30–1.19	0.149
Transfusion	2.81	0.96–8.17	0.058

Abbreviations: HR: Hazard Ratio; 95% CI: Confidence interval (lower bound–upper bound).

## Data Availability

The data presented in this study are available on request from the corresponding author with permission of the responsible institutional review board due to ethical and data protection restrictions.
